# Cerebral artery signal intensity gradient from Time-of-Flight Magnetic Resonance Angiography and clinical outcome in lenticulostriate infarction: a retrospective cohort study

**DOI:** 10.3389/fneur.2023.1220840

**Published:** 2023-09-20

**Authors:** Chan-Hyuk Lee, Jong-Won Chung, Hyung Seok Guk, Ji Man Hong, Robert S. Rosenson, Seul-Ki Jeong

**Affiliations:** ^1^Department of Neurology, Asan Medical Center, Seoul, Republic of Korea; ^2^Department of Neurology, Ulsan University Hospital, University of Ulsan College of Medicine, Ulsan, Republic of Korea; ^3^Department of Neurology, Samsung Medical Center, Seoul, Republic of Korea; ^4^Department of Neurology, Gunsan Medical Center, Gunsan, Republic of Korea; ^5^Department of Neurology, Ajou University School of Medicine, Suwon, Republic of Korea; ^6^Mount Sinai Heart, Icahn School of Medicine at Mount Sinai, New York, NY, United States; ^7^Seul-Ki Jeong Neurology Clinic, Seoul, Republic of Korea

**Keywords:** cerebral artery, functional outcome, lenticulostriate infarction, signal intensity gradient, time-of-flight magnetic resonance angiography, wall shear stress

## Abstract

**Purpose:**

Lenticulostriate infarction requires further research of arterial hemodynamic factors, as the disease is diagnosed in the absence of major arterial stenosis or cardioembolism.

**Methods:**

In this multicenter retrospective cohort study, we included patients who were hospitalized for lenticulostriate infarction from January 2015 to March 2021 at three stroke centers in South Korea. We obtained hemodynamic information on cerebral arteries using signal intensity gradient (SIG), an *in-vivo* approximated wall shear stress (WSS) derived from Time-of-Flight Magnetic Resonance Angiography (TOF-MRA). A favorable outcome was defined as a modified Rankin Scale of 0 to 2 at hospital discharge.

**Results:**

A total of 294 patients were included, of whom 146 (49.7%) had an unfavorable outcome. The unfavorable outcome group showed significantly lower SIG in both middle cerebral arteries (MCAs) than the favorable group (5.2 ± 1.2 SI/mm vs. 5.9 ± 1.2, *p* < 0.001), and similar findings were observed in other cerebral arteries. The SIGs in both MCAs were independently associated with favorable outcome, with an odds ratio of 1.42 (95% confidence interval, 1.11–1.80; *p* = 0.005) for the right MCA and 1.49 (95% CI, 1.15–1.93; *p* = 0.003) for the left MCA, after adjusting for potential confounders. Similar findings were observed in other cerebral artery SIGs.

**Conclusion:**

Cerebral artery SIG from TOF-MRA was significantly associated with short-term functional outcomes in patients with lenticulostriate infarction. Further studies are needed to investigate the temporal relationships of SIG in patients with cerebral infarction.

## 1. Introduction

Arterial wall shear stress (WSS) is a critical determinant of endothelial homeostasis, exerting a frictional force along the arterial tree (1). While laminar flow predominates in straight arterial segments, it is disturbed in branches and curves, resulting in vortices and eddy currents and causing low or recirculating WSS. Disturbed flow is known to contribute to arterial pathologies such as atherosclerotic stenosis or aneurysmal dilatation (2), which can result in stroke, a leading cause of disability that compromises a patient’s quality of life (3).

However, the association between disturbed flow and arterial pathology is not straightforward in certain categories of ischemic stroke. Lacunar infarction or lenticulostriate infarction, for example, are diagnosed in the absence of underlying arterial pathologies or cardiac abnormalities. Lacunar infarction occurs in subcortical white matter and is classified as small vessel occlusion (SVO), although the underlying pathophysiology remains incompletely understood. Previous studies using ultrasonography in carotid arteries have shown that patients with lacunar infarction exhibit significantly lower WSS than controls (4).

Compared to lacunar infarction, lenticulostriate infarction occurs in the territory supplied by the deep perforating branches of the middle cerebral artery (MCA) or anterior cerebral artery (ACA) (5). Its etiology has been reported to include cardiac embolism (6), lacunar infarction (7), or branch atheromatous disease (8, 9). Lenticulostriate infarction involves the condensed white matter tracts in the basal ganglia or surrounding subcortical regions, and a significant number of patients require lifelong assistance for daily living (10). Despite its impact, studies on the clinical prognosis and WSS in lenticulostriate infarction are lacking.

In this study, we hypothesized that acute-stage arterial WSS might be associated with prognosis in lenticulostriate infarction. Because arterial WSS is a force acting on the endothelium that is shared among cerebrovascular networks without underlying stenosis, we measured arterial wall signal intensity gradient (SIG), an approximated WSS derived from an interpolation of discrete space (pixels) from Time-of-Flight Magnetic Resonance Angiography (TOF-MRA) (11). Arterial wall SIG was measured for all cerebral arteries concurrently using TOF-MRA in the acute stage of infarction. Arterial wall SIG has been shown to reveal hemodynamic features in various vascular diseases, including moyamoya disease (12) and ischemic stroke of large artery atherosclerosis (13). We conducted a retrospective analysis of patients with lenticulostriate infarction from three nationwide stroke centers, collecting clinical data, including modified Rankin Scale (mRS) at discharge.

## 2. Materials and methods

### 2.1. Study population

This is a multicenter retrospective cohort study of consecutive patients hospitalized for acute ischemic stroke in the lenticulostriate artery (LSA) territory between January 2015 and March 2021. Patients were registered at one of the three centers: Jeonbuk National University Hospital in Jeonju, Samsung Medical Center in Seoul, and Gunsan Medical Center in Gunsan.

The inclusion criteria were as follows: (1) patients aged 18 years or older who underwent brain MRI including diffusion-weighted imaging (DWI) and apparent diffusion coefficient for acute ischemic stroke within 3 days of ischemic stroke, (2) patients who underwent cerebral angiographic measurements that included TOF-MRA for cerebral arteries, and (3) patients with high signal intensity lesions in the unilateral LSA territory on DWI. Patients who fulfilled the inclusion criteria were designated as having a lenticulostriate infarction and included in the present study.

The exclusion criteria were as follows: (1) patients with moderate-to-severe (>50%) stenosis or occlusion of the major intracranial or extracranial arteries, including internal carotid artery (ICA), MCA, ACA, posterior cerebral artery (PCA), basilar artery (BA), and vertebral artery (VA), and (2) patients with ischemic stroke due to cardiac or other determined etiology (e.g., arterial dissection, moyamoya disease).

### 2.2. Acquisition of clinical data

Demographic and clinical information on cardiovascular risk factors, such as hypertension, diabetes mellitus, hyperlipidemia, and smoking history, as well as laboratory findings at admission, including complete blood count, lipid profiles, serum glucose level, and HbA1c, were extracted from electronic medical records. Neurological severity was assessed using the National Institute of Health Stroke Scale (NIHSS) and modified Rankin Scale (mRS) during hospitalization. Ischemic lesions were categorized according to the involvement of the basal ganglia or internal capsule. To compare lesion size, the ischemic lesion-to-whole brain volume ratio was calculated using three-dimensional segmentation methods as previously reported (14).

### 2.3. Clinical outcome

For patients with lenticulostriate infarction, the clinical outcomes were dichotomized by the mRS score measured on the 7th day or at discharge if the patient was hospitalized for less than 7 days. A favorable outcome was defined as an mRS score of two or less, while an unfavorable outcome was defined as an mRS score of three or more.

### 2.4. Time-of-flight magnetic resonance angiography

The Time-of-flight magnetic resonance angiography (TOF-MRA) protocols used in the three hospitals were described in detail, and the data are provided in Supplementary Table 1. In brief, all three hospitals used a 3.0 T MR of 32 channels cardiac coil (Achieva or Ingenia Cx, Philips Medical Systems, The Netherlands) to obtain a three-dimensional intracranial and extracranial TOF image. The following parameters were used to obtain TOF-MRA: repetition time (TR) of 20.0–25.0 ms, echo time (TE) of 3.45–3.50 ms, and flip angle (FA) of 20.0°.

### 2.5. Measurement of signal intensity gradient of cerebral arteries

TOF-MRA signal intensity gradient (SIG), as a surrogate marker of WSS, was measured in cerebral arteries (both ICA, MCA, ACA, PCA, VA, and single BA) with a semi-automated software (VINT, Mediimg, Inc. Seoul, Republic of Korea) as reported previously (11). In brief, the signal intensities at the iso-point (*Φ_a_; SI at position A* [Xa] *along the arterial contour line*) and at the inner point (*Φ_b_; SI at position B* [Xb]) were calculated by using a trilinear interpolation algorithm based on the positions and signal intensities in the eight neighboring voxels. The signal intensities of TOF-MRA were normalized to eliminate the offset and scale effects across the MRA datasets. For each iso-point (position A), the SIG was calculated from the difference in signal intensities between points A and B as follows:


(1) $$\text{SIG},\text{SI}/\text{mm}=\left(\Phi_{b}–\Phi_{a}\right)/│X_{b}–X_{a}│$$

**Figure 1:**
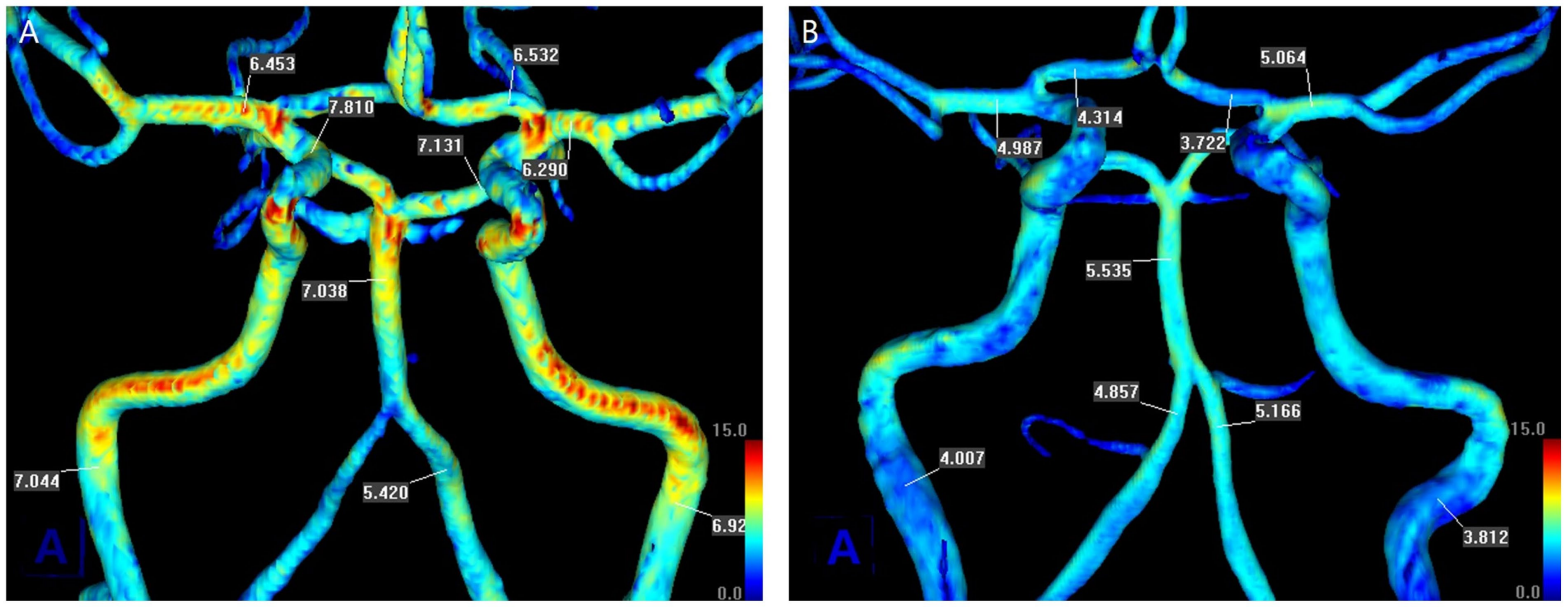
Measurement of the mean values of signal intensity gradient (SIG) of cerebral arteries from TOF-MRA in two representative patients. There was no underlying stenosis in the cerebral arteries. **(A)** A 67-year-old man with left lenticulostriate infarction, whose modified Rankin Scale (mRS) score was 3 on admission and 1 at discharge. **(B)** A 60-year-old woman with right lenticulostriate infarction, whose mRS score was 3 on admission and at discharge. Each scale bar is the same in magnitude.

We obtained the mean SIG of major cerebral arteries as shown in two representative patients (Figure 1). The arterial SIG was measured in the relatively straight segment to select the arterial segments with laminar flow (15). For the ICA, the C1 distal portion before the horizontal intrapetrous segment was selected, while the V4 distal portion just before the conjoined BA was selected for the VA. For the BA, the mid-to-distal portion was chosen. For the MCA and the ACA, the proximal 1/2 or 1/3 segment was chosen, while the P2 segment (distal to the posterior communicating artery) was selected for the PCA.

### 2.6. Statistical analysis

Descriptive data for the clinical characteristics and laboratory findings of the participants were expressed as mean ± standard deviation (SD) or percentage, as appropriate. The Kolmogorov–Smirnov test was used to assess the normality of distribution. Student’s t-test or chi-squared test was used to assess differences as appropriate. Logistic regression analyses were performed to determine the independent association between the SIG values of cerebral arteries and clinical outcomes after adjusting for other possible confounders. Statistical analyses were conducted using IBM SPSS Statistics for Windows, version 20 (IBM Corp., Armonk, NY, United States).

## 3. Results

A total of 294 patients with ischemic stroke in the LSA territory were included in the study, after excluding 12 (3.9%) patients by a consensus between two neurologists (CHL and SKJ). Six had poor image quality, four were ultimately determined not to be in the LSA territory, and two had different etiologies of cerebral infarction confirmed in further evaluation. Among these patients, 146 (49.7%) were classified as having an unfavorable outcome and were functionally dependent on caregivers in routine activities of daily living (Table 1).

**Table 1:** Demographics of patients with lenticulostriate infarction according to modified Rankin Scale (mRS) score.

	Favorable^†^ (mRS 0–2)	Unfavorable^†^ (mRS 3–5)	*p*-value
Number	148	146	
Age, year	64.6 ± 13.0	72.5 ± 10.5	<0.001
Female, %	38.5	59.6	<0.001
Smoking, %	22.3	13.0	0.037
Lesion side, left, %	56.1	55.5	0.917
Hypertension, %	63.5	61.0	0.651
Type 2 diabetes mellitus, %	22.3	21.9	0.938
Hypercholesterolemia, %	32.4	17.1	0.002
**Laboratory findings**			
White blood cell	7,181	7,158	0.934
Hematocrit, %	41.6 ± 4.8	39.2 ± 4.8	<0.001
Glucose, mg/dL	120.1 ± 33.1	137.5 ± 76.9	0.015
HbA1c, %	6.6 ± 5.1	6.3 ± 2.6	0.543
Total cholesterol, mg/dL	182.8 ± 39.4	179.7 ± 42.8	0.523
Triglyceride, mg/dL	164.1 ± 120.0	172.8 ± 183.9	0.638
HDL cholesterol, mg/dL	49.3 ± 14.1	45.7 ± 13.8	0.031
D-dimer, mg/dL	0.4 ± 0.4	1.0 ± 4.4	0.126
Total homocysteine, mg/dL	13.8 ± 9.7	12.5 ± 3.9	0.182
**Hospital progress**			
Admission, days	6.1 ± 7.5	9.4 ± 12.2	0.005
NIHSS, first day	2.7 ± 1.8	4.9 ± 2.2	<0.001
NIHSS, seventh day or at discharge	2.1 ± 1.6	4.9 ± 2.4	<0.001
mRS, first day	2.1 ± 1.0	3.3 ± 0.6	<0.001
mRS, seventh day or at discharge	1.3 ± 0.7	3.3 ± 0.5	<0.001
**Analysis of ischemic brain lesion**			
Basal ganglionic lesion, %	64.8	80.0	0.004
Internal capsular lesion, %	7.6	9.7	0.530
Ischemic lesion volume ratio, %^‡^	0.11 ± 0.13	0.16 ± 0.13	0.004
**Protocol of TOF-MRA**			
Repetition time, ms	23.5 ± 1.4	23.2 ± 0.9	0.031
Echo time, ms	3.5 ± 0.4	3.5 ± 0.0	0.158

Mean ± standard deviation (SD) or percentages shown, as appropriate. *p*-values by *t*-test or Chi-square test, as appropriate. ^†^Clinical outcome of patients with lenticulostriate artery infarction was determined according to mRS scores at discharge or the seventh day after admission. ^‡^Ischemic lesion to whole brain volume ratio. HbA1c, hemoglobin A1c; HDL, high density lipoprotein; NIHSS, National Institute of Health Stroke Scale; mRS, modified Rankin Scale; TOF-MRA, time of flight magnetic resonance angiography.

The unfavorable outcome group was older and had lower percentages of males, smokers, and dyslipidemia compared to the favorable outcome group. The favorable outcome group showed higher levels of hematocrit and high-density lipoprotein cholesterol and lower serum glucose levels. The favorable outcome group had lower NIHSS and mRS scores throughout the admission period and shorter hospitalization periods. The ischemic lesion-to-whole brain volume ratio was higher, and basal ganglionic lesion was more prevalent in the unfavorable outcome group (64.8% vs. 80.0%, *p* = 0.004).

Figure 1 shows a representative patient in each group for cerebral arterial SIG. The patient in the favorable outcome group (Figure 1A), who had an initial mRS score of 3 and a score of 1 at discharge, demonstrated generally higher values of SIG in cerebral arteries than the patient in the unfavorable outcome group (Figure 1B), who maintained an mRS score of 3 from admission to discharge. The unfavorable outcome group showed significantly lower SIG in cerebral arteries than the favorable group (Table 2); in both MCAs, the unfavorable group had a mean SIG of 5.2 ± 1.2 (SI/mm), which was significantly lower than the favorable group (5.9 ± 1.4, *p* < 0.001, respectively).

**Table 2:** Mean values of signal intensity gradient (SIG) from TOF-MRA in cerebral arteries according to clinical outcome.

SIG, SI/mm	Favorable (mRS 0–2)	Unfavorable (mRS 3–5)	*p*-value
Right ICA	7.0 ± 2.1	5.8 ± 1.7	<0.001
Left ICA	6.8 ± 2.0	5.9 ± 1.6	<0.001
Right VA (No^†^)	6.7 ± 1.8 (126)	5.8 ± 1.5 (121)	<0.001
Left VA (No^†^)	6.5 ± 1.7 (135)	5.7 ± 1.6 (129)	<0.001
BA	8.7 ± 2.7	7.2 ± 2.2	<0.001
Right MCA	5.9 ± 1.4	5.2 ± 1.2	<0.001
Left MCA	5.9 ± 1.4	5.2 ± 1.2	<0.001
Right ACA (No^†^)	6.1 ± 1.6 (138)	5.2 ± 1.4 (133)	<0.001
Left ACA (No^†^)	6.0 ± 1.5 (132)	5.2 ± 1.3 (133)	<0.001
Right PCA (No^†^)	6.7 ± 2.0 (133)	5.8 ± 1.8 (130)	<0.001
Left PCA (No^†^)	6.6 ± 1.9 (134)	5.9 ± 1.8 (130)	0.008

Mean ± standard deviation (SD) shown. ^†^Number of examined for SIG of TOF-MRA. *p*-values by *t*-test. SIG, signal intensity gradient; ICA, internal carotid artery; VA, vertebral artery; BA, basilar artery; MCA, middle cerebral artery; ACA, anterior cerebral artery; PCA, posterior cerebral artery.

In both ICAs, the unfavorable outcome group also showed significantly lower mean SIG values than the favorable group (5.8 ± 1.7 vs. 7.0 ± 2.1 for the right ICA; 5.9 ± 1.2 vs. 6.8 ± 2.0 for the left ICA; all *p* < 0.001). According to mRS scores, cerebral artery SIGs were described in Supplementary Table 2. In all cerebral arteries, increases of mRS score were significantly associated with decreases of SIG values.

As all cerebral artery SIGs were significantly different according to clinical outcome in patients with unilateral lenticulostriate infarction, side-to-side SIG comparisons were evaluated according to clinical outcome or lesion side (Table 3).

**Table 3:** Right to left comparison of SIG in cerebral arteries according to outcome or lesion side in the patients with lenticulostriate infarction.

	According to clinical outcome			
	Favorable (mRS 0–2)		Unfavorable (mRS 3–5)	
SIG, SI/mm	Right	Left	Right	Left
ICA	7.0 ± 2.1	6.8 ± 2.0^†^	5.8 ± 1.7	5.9 ± 1.6
VA	6.7 ± 1.8	6.5 ± 1.7	5.8 ± 1.5	5.7 ± 1.6
MCA	5.9 ± 1.5	5.9 ± 1.4	5.2 ± 1.2	5.2 ± 1.2
ACA	6.2 ± 1.6	6.0 ± 1.5^‡^	5.2 ± 1.3	5.2 ± 1.3
PCA	6.7 ± 2.0	6.6 ± 1.9	5.9 ± 1.8	6.0 ± 1.9

	According to lesion side of lenticulostriate infarction			
	Favorable (mRS 0–2)		Favorable (mRS 0–2)	
SIG, SI/mm	Right	Left	Right	Left
ICA	6.2 ± 2.1	6.3 ± 2.0	6.6 ± 1.8	6.4 ± 1.8^‡^
VA	6.0 ± 1.6	5.9 ± 1.7	6.5 ± 1.8	6.3 ± 1.7
MCA	5.4 ± 1.4	5.4 ± 1.3	5.6 ± 1.4	5.7 ± 1.4
ACA	5.5 ± 1.4	5.4 ± 1.4	5.9 ± 1.7	5.7 ± 1.5
PCA	6.2 ± 1.9	6.2 ± 2.0	6.5 ± 2.0	6.4 ± 1.9

Mean ± standard deviation (SD) shown. ^†^*p*-value < 0.05. ^‡^*p* < 0.01 by paired *t*-test.

In the favorable outcome group, significantly lower SIG values were observed in the left ICA and ACA. However, in the unfavorable outcome group, there was no side-to-side difference in arterial SIG. Regarding lesion-sidedness, patients with left-sided lenticulostriate infarction showed lower SIG values in the left ICA (ipsilesional) than the right ICA; however, this finding was not observed in patients with right-sided infarction.

Multivariate logistic regression analyses were performed to examine the association between cerebral arterial SIG and favorable outcomes (i.e., mRS score 2 or less; Table 4).

**Table 4:** Multivariate association between SIGs of cerebral arteries and the favorable clinical outcome (mRS 0–2) in patients with lenticulostriate infarction.

	Favorable outcome (mRS 0–2)		
SIG of arteries, SI/mm	Adjusted OR^†^	95% CI	*p*-value
Right ICA	1.34	1.13–1.59	0.001
Left ICA	1.24	1.04–1.48	0.017
Right VA	1.29	1.05–1.60	0.017
Left VA	1.25	1.03–1.52	0.022
BA	1.20	1.06–1.37	0.005
Right MCA	1.42	1.11–1.80	0.005
Left MCA	1.49	1.15–1.93	0.003
Right ACA	1.47	1.15–1.86	0.002
Left ACA	1.39	1.08–1.79	0.011
Right PCA	1.14	0.95–1.38	0.157
Left PCA	1.04	0.86–1.25	0.695

^†^Adjusted by age, sex, smoking, hypercholesterolemia, hematocrit, serum glucose, HDL cholesterol, repetition time of TOF-MRA, ischemic lesion volume ratio, and basal ganglionic lesion. SIG, signal intensity gradient; mRS, modified Rankin Scale; OR, odds ratio; CI, confidence intervals; ICA, internal carotid artery; VA, vertebral artery; BA, basilar artery; MCA, middle cerebral artery; ACA, anterior cerebral artery; PCA, posterior cerebral artery.

The SIGs of both MCAs were independently associated with favorable outcome, showing an odds ratio (OR) of 1.42 (95% confidence interval [CI] 1.11–1.80, *p* = 0.005) for the right MCA and 1.49 (95% CI, 1.15–1.93, *p* = 0.003) for the left MCA, after adjusting for potential confounders. Both ICA SIGs also showed significant associations with favorable outcomes, with an OR of 1.34 (95% CI 1.13–1.59, *p* = 0.001) for the right ICA and 1.24 (95% CI, 1.04–1.48, *p* = 0.017) for the left ICA. Other cerebral arteries, including ACAs, VAs, and BA, also showed significant associations with favorable outcomes, while PCAs did not. Among the independent variables, only age was negatively associated with the favorable outcome in all arterial models.

## 4. Discussion

The present study investigated the association between cerebral arterial SIG and clinical outcomes in patients with lenticulostriate infarction. We found that almost half of the patients had an unfavorable outcome (i.e., mRS score 3 or more) at short-term intervals. The main finding of this study was that higher arterial SIG was associated with better clinical prognosis. This association was observed not only in the relevant artery for the lenticulostriate artery, but in nearly all cerebral arteries except for PCAs.

Cerebrovascular networks have well-developed collateral circulations and channels, and the neurovascular status of patients with ischemic stroke is more susceptible to neurohormonal effects compared to healthy individuals (16). This susceptibility can affect the function of brain cells and recovery following ischemic stroke (17). Cerebral arterial resistance is contributed equally by both large arteries and small arterioles (18), but small arterioles (<100 μm diameter) play a major role in vascular resistance in peripheral circulation (19), Dysfunction of the cerebrovascular network regulating blood flow distribution across large arteries can result in global hypoperfusion in the brain parenchyma (20). The association between arterial SIG and clinical outcomes suggest that variable degrees of cerebral hypoperfusion can occur depending on the patient’s vascular network (21).

Previous studies have reported that hypoperfusion surrounding an ischemic core lesion is associated with short- or long-term prognosis, using methods such as a perfusion-CT (22), MRI (23), or Single Photon Emission Computed Tomography (24). However, the perfusion defect in association with prognosis was closely related to the size of the ischemic stroke and underlying arterial diseases, limiting the effect of the perfusion defect in small-sized strokes, which exhibit a low rate of hypoperfusion (25). The present study suggests that arterial flow information may have another impact on prognosis. The significant association between arterial SIG and clinical outcome, even in arteries irrelevant to the lesion (e.g., VA, BA, or contralateral side MCA), is difficult to explain. As TOF-MRA was examined only once in the acute stage of ischemic stroke in the present study, it is unclear whether the different distribution of arterial WSS was a result of acute ischemic stroke or a reflection of individual characteristics. Subsequent studies are needed to repeat the examination of arterial SIG to address this issue.

The association between cerebral blood flow modifications and clinical prognosis has been previously reported. Pharmacologically-induced hypertension (PIH) during focal cerebral ischemia has been attempted to improve cerebral perfusion to the area of reduced blood flow (26, 27). Previous studies have reported that PIH is safe and effective for early neurologic improvement and long-term functional independence in non-cardioembolic stroke patients (28). Similarly, blood pressure and flow augmentation using external counterpulsation have been reported to be effective in ischemic stroke patients with large artery occlusive disease (29), improving cerebral perfusion to the area of reduced blood flow, albeit with global effects.

The prognosis of lenticulostriate infarction largely depends on whether the lesion involves the basal ganglia or the internal capsule. In the present study, which exclusively included patients with lenticulostriate infarction, the distribution of lesions involving the condensed white matter tracts surrounding the internal capsule could be a critical factor for the high proportions of functional dependence (30). While the mechanism of lenticulostriate infarction is not thoroughly understood, the present study suggests that the basal ganglia and its surrounding regions may be more vulnerable to hemodynamic compromise than other brain areas.

The present study has several limitations. First, due to its retrospective design, follow-up TOF-MRA and arterial SIG measurements could not be obtained for patients who have no underlying vascular pathologies. Second, the modality for measuring arterial SIG still needs more validated data for intracranial TOF sequences, especially for MCA, ACA, and PCA. In the distal portion of the artery, signal intensities could be susceptible to intraluminal saturation. Third, the mRS score was measured on the 7th day or less. The 3-month mRS is more suitable for a long-term prognosis. Lastly, clinical outcomes were determined only using mRS scores. NIHSS, another measurement for neurological deficit, seemed to be limited in use as a prognostic indicator in patients with minor subcortical stroke, as the score covers a wide spectrum of neurologic and cortical symptoms.

## 5. Conclusion

In this study, we found a significant association between cerebral arterial SIG measured from TOF-MRA and short-term functional outcomes in patients with lenticulostriate infarction. Our findings show that higher SIG values in cerebral arteries are associated with better clinical outcomes. Further studies are needed to investigate the temporal relationships between SIG and cerebral infarction in various subtypes of ischemic stroke across different ethnicities.

## Data availability statement

The raw data supporting the conclusions of this article will be made available by the authors, without undue reservation.

## Ethics statement

The studies involving humans were approved by the Ethics Review Committee in Jeonbuk National University Hospital (2021-04-056-002) and Samsung Medical Center (2008-02-046-086). The studies were conducted in accordance with the local legislation and institutional requirements. The Ethics Committee/Institutional Review Board waived the requirement for written informed consent from the participants or their legal guardians/next of kin because it was a retrospective study reviewing patient records and images.

## Author contributions

C-HL, J-WC, and S-KJ: conceptualization and funding acquisition. C-HL and S-KJ: methodology. S-KJ, and J-WC: formal analysis and investigation. C-HL and J-WC: writing—original draft preparation. HG, JH, RR, and S-KJ: writing—review and editing. S-KJ: supervision. All authors contributed to the article and approved the submitted version.
