# Supplementary material for: Cerebral artery signal intensity gradient from Time-of-Flight Magnetic Resonance Angiography and clinical outcome in lenticulostriate infarction: a retrospective cohort study
Source: Front Neurol. 2023 Sep 20;14:1220840. doi: 10.3389/fneur.2023.1220840 (PMC10547899; doi:10.3389/fneur.2023.1220840)
Supplement: Supplementary file 1 [file Data_Sheet_1.PDF]

Supplementary Table 1. Protocols of brain 3D Time-of-Flight Magnetic Resonance Angiography (TOF-MRA)

	**Protocols of TOF-MRA**		
	**Gunsan Medical Center**	**Jeonbuk National University Hospital**	**Samsung Medical Center**
Tesla	3.0	3.0	3.0
Repetition time, ms	20.0	23.0	25.0
Echo time, ms	3.45	3.5	3.45
Flip angle, °	20.0	20.0	20.0
Resolution	488×310	300×220	880×310
Field of view	200	180	200
Slices	152	160	152
Manufacturer	Ingenia Cx, Philips Medical System	Achieva, Philips Medical System	Achieva, Philips Medical System

Supplementary Table 2. Mean values of signal intensity gradient (SIG) in cerebral arteries according to modified Rankin Scale (mRS) score

	SIG of cerebral arteries, SI/mm										
**mRS**	Rt ICA	Lt ICA	Rt VA	Lt VA	BA	Rt MCA	Lt MCA	Rt ACA	Lt ACA	Rt PCA	Lt PCA
0	7.4 ± 1.8	7.0 ± 1.8	7.0 ± 1.7	6.8 ± 1.7	8.8 ± 2.4	6.1 ± 1.4	6.0 ± 1.3	6.3 ± 1.7	6.3 ± 1.4	7.3 ± 2.0	7.0 ± 2.2
1	7.4 ± 1.9	7.2 ± 1.9	6.9 ± 1.6	6.6 ± 1.7	9.1 ± 2.7	6.2 ± 1.5	6.1 ± 1.4	6.3 ± 1.6	6.2 ± 1.4	6.8 ± 2.0	6.7 ± 1.9
2	6.6 ± 2.2	6.4 ± 2.1	6.5 ± 1.9	6.3 ± 1.8	8.3 ± 2.8	5.6 ± 1.4	5.7 ± 1.3	5.9 ± 1.6	5.7 ± 1.5	6.5 ± 2.0	6.3 ± 1.8
3	5.7 ± 1.6	5.8 ± 1.6	5.9 ± 1.4	5.7 ± 1.6	7.1 ± 2.2	5.1 ± 1.2	5.2 ± 1.1	5.2 ± 1.3	5.1 ± 1.3	5.8 ± 1.7	5.9 ± 1.8
4	5.8 ± 1.7	6.0 ± 1.6	5.7 ± 1.8	5.7 ± 1.5	7.3 ± 2.4	5.3 ± 1.2	5.3 ± 1.2	5.3 ± 1.4	5.3 ± 1.3	5.9 ± 1.9	6.1 ± 1.8
Overall	6.4 ± 2.0*	6.3 ± 1.9*	6.3 ± 1.7*	6.1 ± 1.7*	8.0 ± 2.6*	5.5 ± 1.4*	5.6 ± 1.3*	5.7 ± 1.5*	5.6 ± 1.4*	6.3 ± 2.0‡	6.3 ± 1.9†

† P < 0.05, ‡ P < 0.01, * P < 001 by analysis of variance (ANOVA).
